# Correctly identifying the cells of origin is essential for tailoring treatment and understanding the emergence of cancer stem cells and late metastases

**DOI:** 10.3389/fonc.2024.1369907

**Published:** 2024-04-10

**Authors:** Helge Waldum, Geir Slupphaug

**Affiliations:** Department of Clinical and Molecular Medicine, Faculty of Medicine and Health Sciences, Norwegian University of Science and Technology, Trondheim, Norway

**Keywords:** bone metastases, cancer cell of origin, cell adherence, differentiated versus stem cell, direct versus indirect stimulation, dormancy, late metastases

## Abstract

Malignancy manifests itself by deregulated growth and the ability to invade surrounding tissues or metastasize to other organs. These properties are due to genetic and/or epigenetic changes, most often mutations. Many aspects of carcinogenesis are known, but the cell of origin has been insufficiently focused on, which is unfortunate since the regulation of its growth is essential to understand the carcinogenic process and guide treatment. Similarly, the concept of cancer stem cells as cells having the ability to stop proliferation and rest in a state of dormancy and being resistant to cytotoxic drugs before “waking up” and become a highly malignant tumor recurrence, is not fully understood. Some tumors may recur after decades, a phenomenon probably also connected to cancer stem cells. The present review shows that many of these questions are related to the cell of origin as differentiated cells being long-term stimulated to proliferation.

## 1 Introduction

Malignancy is characterized by deregulated growth and the ability to invade surrounding tissues or metastasize to other organs. Current treatments for malignant tumors include surgery, irradiation, and cytotoxic drugs, often leading to the apparently complete removal of tumors. Unfortunately, many such tumors recur with reduced treatment susceptibility, even after initially effective interventions. Such recurrence often leads to the death of the patient and has been explained by the dormancy of malignant cells that initially survived the cytotoxic treatment (1). The mechanisms governing entry into and emergence from dormancy remain inadequately understood (1). The present review aims to elucidate this phenomenon with a particular emphasis on the cells of origin of cancers.

## 2 Cell of origin: differentiated versus stem cells

Our points of view are mainly based upon long-term experience in gastroenterology including gastric physiology and pathology. During the late seventies and early eighties, it became evident that the gastric hormone gastrin stimulated the enterochromaffin-like (ECL) cell to proliferation and neoplasia in rodents and humans (2–5), and before the identification of the ECL cell, Azzopardi and Pollock (6) focused on argentaffin (neuroendocrine cell marker) cells in gastric carcinomas. The description of ECL cell carcinoids in rodents after long-term profound acid inhibition (7, 8) made the gastrin hypothesis accepted (9). Curiously, although the use of proton pump inhibitors (PPIs) in clinical doses induced hypergastrinemia and ECL cell hyperplasia (10), it was claimed that in humans, unlike animals, hypergastrinemia did not transform ECL cells into cancer (11). Surprisingly, only our group started to examine gastric cancers for ECL cell markers. Our initial publication revealed ECL cell differentiation mainly in diffuse-type gastric carcinomas (12). A critical comment from Creutzfeldt and Solcia (13) was addressed, demonstrating that the criticism was unfounded (14). Continuing our investigation into the role of ECL cells in gastric carcinogenesis, we collaborated with leading pathologist Julia Polack, validating our initial results through thorough immuno-histochemical methods (15). Employing immunoelectron microscopy to demonstrate neuroendocrine granules (16) and *in situ* hybridization to detect expression of the gastrin receptor on gastric cancer cells (17–19), we substantiated that the ECL cell was central in gastric carcinogenesis. Moreover, we tracked the progression of an ECL cell carcinoid to a highly malignant cancer (20) and the evident transformation potential of long-term trophic stimulation of differentiated cells, leading to the development of highly malignant tumors. Similarly, the inactivation of the von Hippel-Lindau factor, whether hereditary or sporadic, results in the activation of hypoxia-inducible factor 2α (HIF-2α) (21), which chronically stimulates the erythropoietin (EPO) cell, being the cell of origin of the most prevalent type of renal cancer (clear cell renal cell cancer) (22). Moreover, the progression to pituitary carcinoma after bilateral adrenalectomy may also be seen as a consequence of long-term growth stimulation (23) like the occurrence of postmenopausal estrogen receptor-positive breast cancer after sex steroid hormone treatment (24).

Another interesting aspect lies in understanding why certain cells are more prone to develop into malignancy. For instance, the ECL cell, characterized by its lack of expression of the adherence molecule E-cadherin (25), may be particularly predisposed to malignancy. This is evident in hereditary gastric cancer, where mutations in the CDH1 gene result in the absence of E-cadherin, ultimately leading to gastric cancer (26). The temporal relationship between hypergastrinemia and the development of malignancy was demonstrated by a Spanish family with a missense mutation in the gene encoding the catalytic subunit of the gastric H^+^/K^+^-ATPase pump (ATP4A), which transports H^+^ ions in exchange for K^+^ ions across the apical membrane of parietal cells. Individuals with this mutation developed tumors of varying malignancy in their twenties and thirties (27). Accordingly, it can be concluded that differentiated cells may progress to malignancy through long-term stimulation of proliferation (20, 27) *via* phases of hyperplasia to benign neoplasia to malignant tumors in the stomach, but probably also in other organs. This process unfolds over extended periods, often spanning decades. Cells with proliferative capacity and low adherence to neighboring cells like the diffuse neuroendocrine (NE) cells are especially prone. Carcinogenesis is a multistage process, and therefore, cells of varying malignancy may co-exist. During the early stages, cells with low adherence and even low proliferation may nevertheless metastasize. These cells, which in many aspects resemble normal cells, may not readily respond to cytotoxic treatment but gradually accumulate new mutations leading to increased malignancy and subsequently manifest as late metastases. Tumors belonging to the phaeochromocytoma/paraganglioma group show histology close to normal, making it impossible to predict malignancy (28). In fact, only metastases that may occur decades after initial diagnosis (29) can definitively determine whether these tumors were benign or malignant. Unfortunately, there are no studies investigating the expression of E-cadherin or other adherence molecules in neuroendocrine cells except the ECL cell (25), but the fact that they appear intermingled between other epithelial cells must imply that they have low cell-to-cell adhesion. Low cell-to-cell adhesion secondary to changes in the E-cadherin–catenin complex has also been focused on in gastric carcinogenesis (30).

The enterochromaffin (EC) cell in the small intestine (SI) can also develop into neuroendocrine tumors (SI-NETs), but in contrast to ECL cell-derived tumors, the pathogenesis of EC cell SI-NETs is not known. Nevertheless, they probably play a role in the development of SI adenocarcinomas (31). Neuroendocrine tumors (NETs) in the small intestine are the most prevalent tumors in that organ. Interestingly, such tumors may develop in clusters, and they most often lack driver mutations (32), indicating a local carcinogenic factor.

The ability to develop into malignant tumors is not restricted to differentiated cells presently accepted as neuroendocrine but probably also other proliferating cells with low adherence. Moreover, more tumors develop from NE cells than presently recognized. For instance, we have shown that the most prevalent type of kidney cancer, clear cell renal cell cancer (CCRCC), most likely originates from EPO cells (21, 22, 33). Clinically, these tumors exhibit similarities to NETs, including early metastases, low response to cytotoxic drugs, and, parallel to hormonal overstimulation in NETs, erythrocytosis in a subset of patients. They also share positivity for neuron-specific enolase (NSE) (33, 34). NSE is a more specific neuroendocrine marker than ordinarily believed (34). It is probably the most sensitive neuroendocrine marker, which has also contributed to its dubious reputation as non-specific. Hypoxia-inducible factor (HIF) is the most important regulator of erythropoietin release (35). Von Hippel-Lindau (VHL) disease predisposes to CCRCC by stabilization of HIF, thereby leading to increased and continuous stimulation of the EPO cell, both functionally and proliferatively (36). Lack of VHL factor function is also central in CCRCCs in general (37, 38), but the cancers not being a part of inherent VHL disease manifest themselves a few decades later. The prolonged hyperstimulation by HIF causing CCRCC resembles how excessive gastrin hyperstimulation leads to gastric cancer and is another example of cancer developing from a differentiated cell exposed to prolonged hyperstimulation. This improved understanding of the pathogenesis of CCRCC has already resulted in potentially improved treatments such as using HIF inhibitors (36).

Lung cancer is another prevalent cancer where NE cells play an important role in carcinogenesis since small cell and large cell pulmonary cancers are regarded as NE tumors (39). In fact, the prevalence of the more benign variant, NETs, localized to the lungs, is secondary only to the gastrointestinal tract. There are many types of NE cells in the lungs either spread in the mucosa (40) or as collections as neuroendocrine bodies (41) often localized to bifurcations of the airways. The latter type plays an important function in the regulation of blood oxygenation (42). The diffusely spread NE cells in the airway surface are also believed to be regulatory, but their precise functions remain to be elucidated. Lung NE cells are the source of the presently called NETs, previously carcinoids (43), whereas the cell of origin of the small and large cell pulmonary carcinomas remains unknown and is not clarified since the early stages of these tumors have not been identified (44). Finally, trans-differentiation may be an alternative for NE cells as the cell of origin for lung NE cancers as well as cancers of the prostate (45).

## 3 Breast and prostate cancers

Prolonged hormonal stimulation is central in the pathogenesis of breast and prostate cancers, which both probably originate from differentiated cells. Unfortunately, there has been no definitive identification of the specific cells of origin for cancers in these two organs. Both breast and prostate cancers exhibit significant heterogeneity (46, 47). Interestingly, there are similarities regarding cells of origin, whether basal or luminal cells (48, 49). Tumors are also categorized based on their expression of sex hormone receptors, estrogen receptor (ER) (48, 50), or androgen receptor (AR) (49). The central role of estrogen in mammary carcinogenesis is underscored by the large contrast in cancer incidence between the two sexes (51).

**Figure 1:**
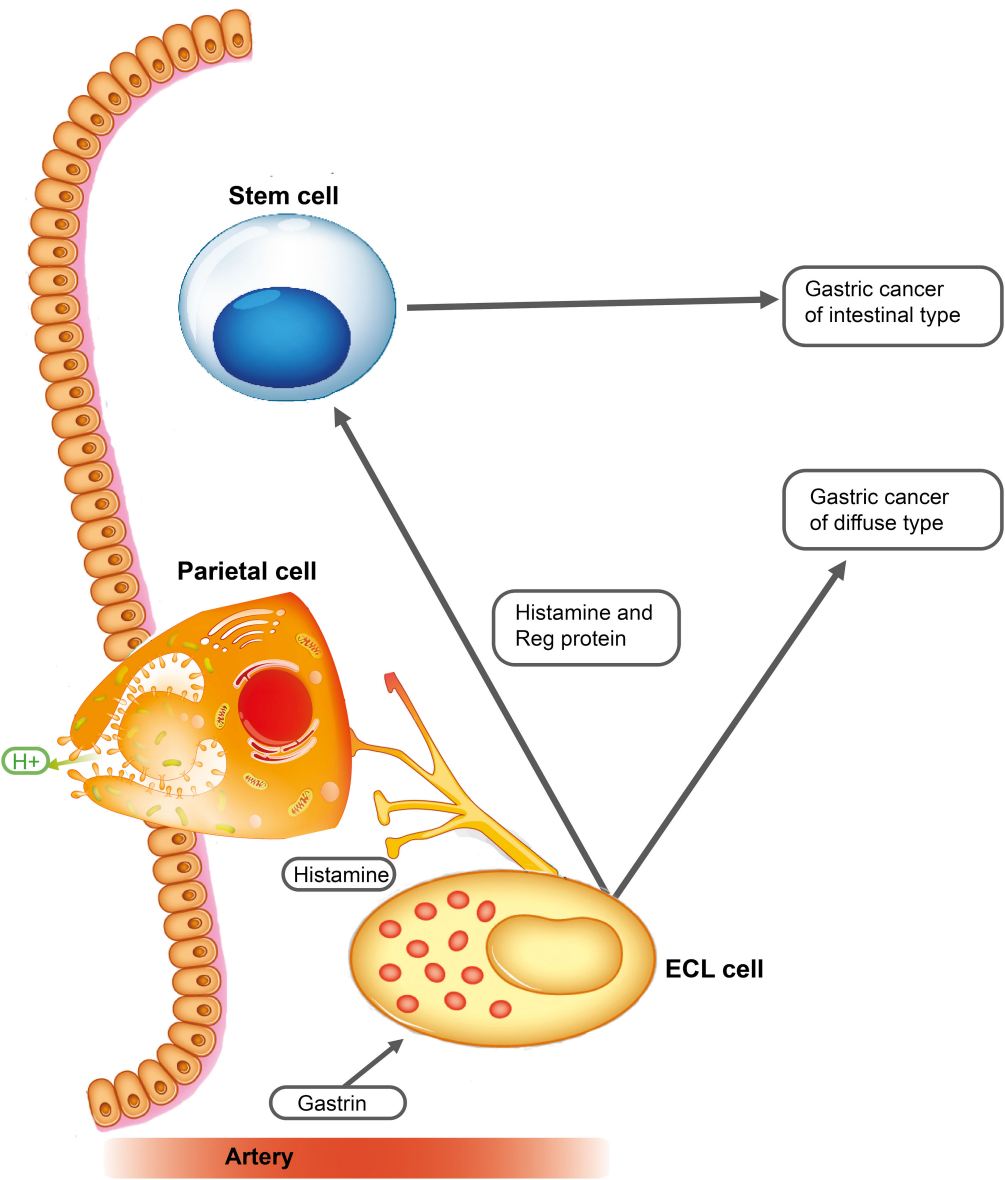
Gastrin stimulates the enterochromaffin-like (ECL) cell to proliferate and to release mediators including regenerating gene (REG) proteins, a family of small proteins with many functions (54), including stimulation of stem cell proliferation (55). Long-term stimulation of these two cells leads to gastric cancers of diffuse and intestinal types [with permission from Waldum et al. (53)].

Although sex hormones are central in the pathogenesis of both breast and prostate cancers, many of these cancers do not express sex hormone receptors. This could be due to mutations during the carcinogenic process or that the tumor nevertheless developed from a sex hormone receptor-negative cell. Thus, while AR was found to be expressed in virtually all luminal cells, half of the basal cells, and 60% of fibroblasts in the normal prostate, there was a loss of AR particularly in the fibroblasts and the tumor cells during the evolution of cancer (52). Another explanation may be that a receptor-positive cell may itself develop into the tumor, and simultaneously, it may provoke neoplasia in another cell (receptor-negative) type by releasing a signal substance-stimulating secretion and proliferation finally leading to neoplasia in this cell as well. This would be parallel to gastric cancers, where gastrin stimulates the proliferation of ECL cells leading to ECL cell NETs and gastric carcinoma of diffuse type. Concomitantly, the release of signal substances from the ECL cell stimulates the proliferation of stem cells (gastrin receptor negative) causing cancer of intestinal type (53) (**Figure 1**).

Sex hormone-induced breast and prostate cancers may promote tumorigenesis by stimulating the proliferation of their target cells, which by releasing signal substances stimulates the growth of another cell type leading to a hormone receptor-negative cancer. Furthermore, overexpression of the chemokine CXCL12-y in prostate and breast epithelial cells induces a shift from a luminal cell type to a non-luminal cell type (56). Among candidates for signal-producing cells in the prostate is the recently identified PEG10+ NE cell (57). NE cells are found in most prostate cancers. However, among the serum markers for NE cells tested, only chromogranin A (CgA) was positively correlated to the number of CgA-staining cells in the tumors (58). Recently, neuropeptide Y was reported to be increasingly expressed during the tumorigenic process in the prostate (59) and thus is a possible driver of carcinogenesis. In families with hereditary increased risk of prostate cancer, there is also an increased risk of breast, gastric, and renal cancers (60). Like in the gastric oxyntic mucosa, there are NE cells located basally in the glands in the prostate (61), whereas this is debatable in the breast (62, 63). Electron microscopy has revealed intracellular secretory granules in prostate NE cells, distinguishing between open-type cells connected to the gland lumen and closed type cells lacking such connection (61, 64). NE cells in the normal prostate cannot be identified by histochemistry using hematoxylin and eosin but with immune histochemistry using traditional markers like chromogranin, synaptophysin, and NSE. Prostate NE cells do not express the androgen receptor or prostate-specific antigen (PSA), and based upon negativity for Ki67, they are also claimed to be non-proliferative (61, 64). Human ECL cells, which for years were claimed not to divide (65), showed a very slow proliferation in that capturing these scarce cells in the act of proliferation was challenging (66). Nevertheless, the lineage plasticity of prostate carcinomas from adenocarcinomas to neuroendocrine androgen receptor-negative cancers represents a major change in cancers, worsening prognosis, and may be due to epigenetic differences (67).

Distinct NE differentiation is evident in various well-defined conventional-type breast carcinomas, but there is still a controversy about whether true NE neoplasms originating from NE cell precursors exist (63). CgA-positive cells have been described in both normal breast tissue and carcinomas (68, 69). Furthermore, by combining immunohistochemistry with gene expression analysis, the percentage of tumors being positive for NE markers increased (70). Breast NE cell carcinoma phenotypes can also be clear cell carcinoma (71), mucinous carcinoma (72), or small cell carcinoma (73). The distinction between luminal and basal cell breast cancers is not definitive, as there is a lineage plasticity where luminal tumors with low ER change in the direction of basal cells (74). The shared reliance on sex hormones in carcinogenesis as well as the parallel mechanisms involved underscores the significant similarities between breast and prostate cancers.

## 4 Metastases

Differentiated cells with poor adherence to each other or supportive tissue cells, such as NE cells interspersed among other cells, are probably more prone to lose connections and disseminate, that is, to metastases. Thus, not only neuroendocrine carcinomas but also the more benign variant NETs often show metastases at an asymptomatic stage (75, 76). This pattern is akin to CCRCCs (77), which most likely also are of neuroendocrine origin (21, 22, 33). Melanoma, originating from melanocytes derived from neural crest cells, also demonstrates early metastasis as well as cellular plasticity (78). This underscores the broader pattern that many cancers develop from differentiated cells (79). Additionally, endometriosis, which is treated like a non-neoplastic benign disease, nevertheless, can metastasize (80). The central role of cellular adherence in carcinogenesis is exemplified by hereditary diffuse gastric cancer, resulting from inactivating mutations in the E-cadherin gene (81). Also, breast cancer (82, 83) and prostate cancer (84) exhibit early metastasis. Thus, these two cancers, being among the most prevalent in women and men, may often have metastasized at diagnosis. Breast cancer screening programs may hopefully increase the rate of diagnosis before metastasis (85), and the new improved methods in the diagnosis of prostate cancer (86) may also improve the prognosis of this cancer.

Nonetheless, the prevailing theory of carcinogenesis is that most cancers develop from stem cells (87), characterized by their rapid proliferation and plasticity. Accumulated evidence suggests that this is an oversimplification. For instance, gastric cancer can develop from an ECL cell, a differentiated NE cell leading to diffuse type cancers, or from stem cells, causing the intestinal type of gastric cancer (53). Although multiple stem cell receptors have been identified (88), their roles in carcinogenesis have received less attention, possibly due to their widespread effects, in contrast to the specific hormone receptors that play a central role in carcinogenesis from differentiated cells. Consequently, the knowledge of the cell of origin as a differentiated cell is presently more useful in tailoring cancer treatment.

## 5 DNA damage resistance in recurrent cancer cells

DNA damage-inducing therapies are fundamental in cancer treatment, as evidenced by the crucial roles of chemotherapy and radiotherapy, both of which operate by directly or indirectly inflicting DNA damage (89). These therapies can alter the DNA nucleotides, induce single-strand (SSBs) or double-strand breaks (DSBs), intercalate between bases, or form crosslinks within DNA or between DNA and proteins (90–94). Additionally, certain compounds induce DNA damage indirectly, e.g., by inhibiting the synthesis of deoxyribonucleotides (95, 96). Cancer cells may develop mechanisms to increase their resistance against DNA-damaging agents, including i) increased efflux or altered metabolism of genotoxic compounds, ii) suppression of apoptosis, or iii) enhanced DNA repair.

Cells harbor elaborate mechanisms to counteract various types of DNA damage, collectively referred to as the DNA damage response (DDR). The DDR is not a rigid pathway but rather a dynamic collection of pathways that are mobilized based on various factors such as type of damage, cell type, chromatin environment, and cell cycle phase (97). Examples of these pathways include base excision repair (BER), which corrects small lesions and certain mismatches (98); ribonucleotide excision repair (RER), which removes mis-incorporated ribonucleotides (99); nucleotide excision repair (NER), which is employed to eliminate bulky and helix-distorting adducts (100); and mismatch repair (MMR), which corrects mismatches generated mainly during replication (101). In the case of DSBs, cells employ either high-fidelity homologous recombination (HR) or the more error-prone non-homologous end-joining (NHEJ) pathway (102, 103). Importantly, the boundaries between these pathways are flexible, and the repair of certain lesions often involves proteins from multiple pathways (104). Additionally, the DDR includes mechanisms to arrest the cell cycle. This is important since DNA-damaging agents primarily exert their cytotoxic effects during the S-phase, and quiescent cells exhibit greater resistance to DNA-damaging drugs (105).

DNA repair plays a crucial but complex role in stem cells. It safeguards normal stem cells and ensures the preservation of genetic integrity as tissues regenerate. Efficient DNA repair mechanisms are particularly prominent in normal stem and progenitor cells. However, as cells differentiate, the tolerance for somatic mutation increases, leading to a reduction in DNA repair activity (106, 107). Enhanced repair of the lesions induced by a specific DNA-damaging agent probably increases resistance to that agent in most cells, including normal and cancer stem cells. However, there are exceptions to this. For example, bone marrow myeloid progenitor cells derived from mice defective in the 3-methyladenine DNA glycosylase Aag exhibit unexpected resistance to alkylation in comparison to progenitor cells from wild-type mice (108). The underlying reason for this is probably that repair intermediates in the BER pathway (AP sites and SSBs) may be more cytotoxic than the original base lesion it left unprocessed. Thus, balanced levels of the factors contributing within that pathway and the availability of redundant repair mechanisms will affect the overall outcome.

The activity of a distinct repair pathway must also be evaluated in relation to the timeframe available for repairing the DNA damage. Even if the expression of many DNA repair proteins is cell cycle regulated and displays elevated levels in rapidly proliferating cells (109), DNA repair processes also take place in quiescent cells. However, the repair pathways employed may differ between these two situations. One example of this is in the repair of DSBs, the most deleterious form of DNA damage. Error-free repair of DSBs *via* HR depends on the presence of an intact copy of the DNA in a newly replicated sister chromatid and thus cannot occur in post-mitotic quiescent cells. Conversely, error-prone NHEJ operates throughout the cell cycle and is active in quiescent cells, including stem cells (110, 111). This shift in repair mode may potentially contribute to increased genomic instability within dormant cancer cells, thereby promoting cancer recurrence by enhancing drug resistance and metastatic potential.

## 6 Cancer stem cells

Cancer stem cells (CSCs) (112) are a subpopulation of cells within a tumor with the ability to self-renew and generate the diverse cell types found in the tumor. They are often considered responsible for tumor initiation, maintenance, and resistance to therapy (113). The proliferative activity of CSCs varies depending on the type of cancer and the specific characteristics of the CSC population. While some CSCs proliferate rapidly and are sensitive to chemotherapy, others proliferate slowly and are more resistant to such treatments, although there is no experimental evidence that CSCs may undergo cell cycle arrest. Thus, dormant cancer cells and CSCs should be considered separate entities (114). Nevertheless, the link between tumor dormancy and CSCs has long been recognized (115) along with the significance of such cells in driving metastasis (116). Although the concept of CSCs initially emerged from observations in acute leukemia (112), these cells have been mainly attributed to cancers originating in cells needing long-term proliferative hyperstimulation before reaching malignancy. Examples include NE cells (different hormones), EPO cells (HIF) in CCRCC, and the cells of cancer origin in the breast (estrogen), the prostate (androgens), and melanocytes (ultraviolet irradiation). Interestingly, in a recent study comparing the genomes at an early stage of cancer with metastatic lesions, the differences were particularly marked for cancers of the prostate, thyroid, CCRCC, breast, and pancreatic NE tumors (116), all belonging to cancers known to lead to late metastases. Tumor cells may be identified by examining bone marrow or blood for specific markers including nucleic acids (113). Comparing mutations in tumor cells with those in primary tumors, early and late metastases will allow an estimation of the stage at which the different tumor cells separated from each other and thus give an indication of the mechanism behind the occurrence of late metastases (117).

## 7 Late metastases/dormancy

**Figure 2:**
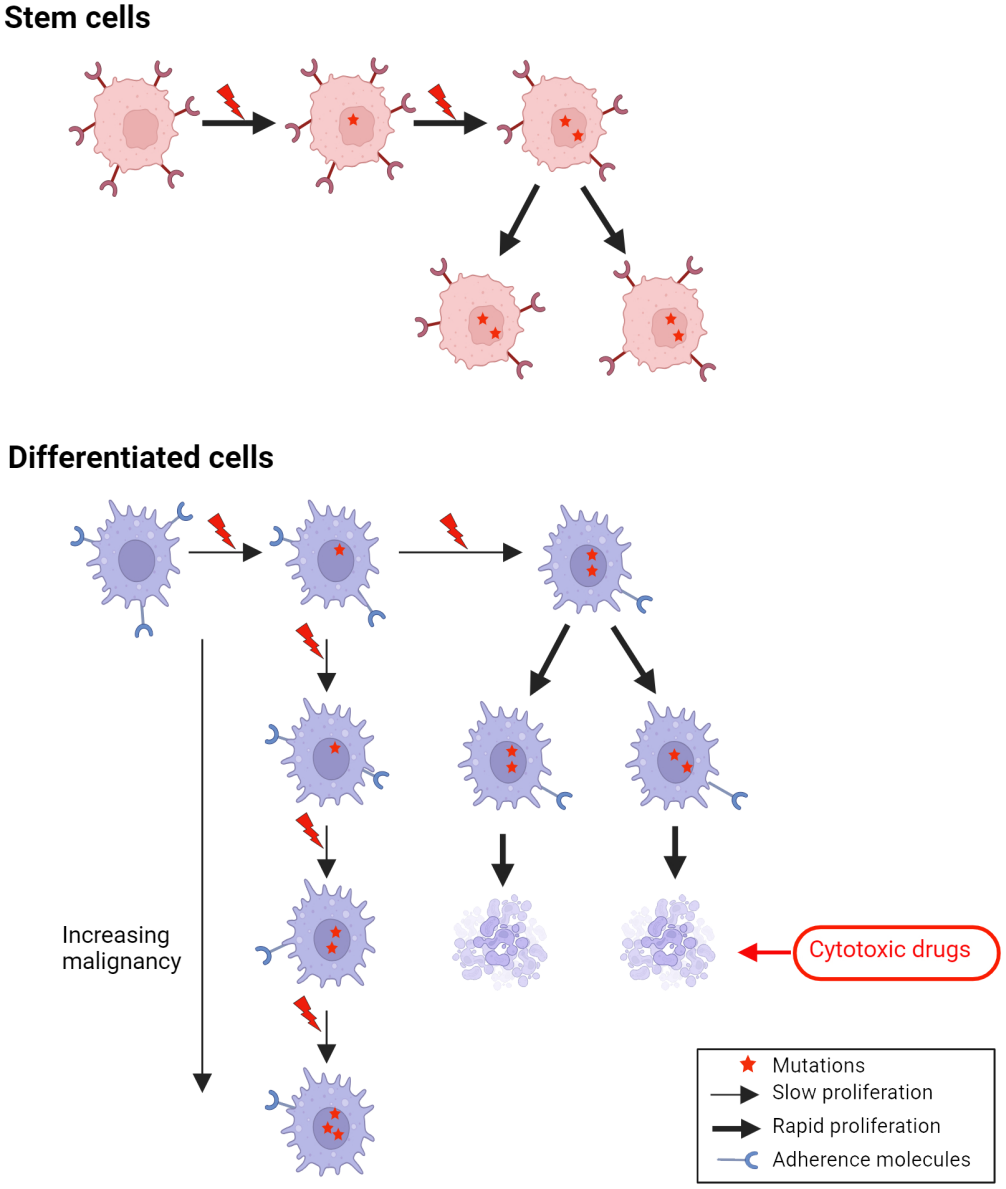
A missense mutation in a gene involved in adherence in differentiated cells, which normally have low adherence, may lead to metastases of cells with rather slow proliferation, which may need years to develop into a clinical tumor (late metastases).

Late metastases will accordingly be expected to occur in cancers originating in differentiated cells with a slow proliferation and little adherence to neighboring cells (**Figure 2**).

Moreover, long-term stimulation of proliferation of the cell of origin mainly by circulating hormones or paracrine substances is often involved. Another peculiar trait of these cancers is metastases to bones (118–124). Even NE tumors, previously thought rarely to metastasize to bones, now are realized to do that often (125). The recognition of bone metastases in NE tumors may be a consequence of increased survival as well as better diagnostic tools for detecting bone metastases. Tumor cell dormancy seems to occur particularly in the bone marrow where both osteoblasts and osteoclasts play a role (125, 126). Late metastases are connected to tumor cell dormancy. The common traits of cancers showing late metastases are summarized in **Table 1**. In a mouse study where prostate cancer tissue was injected subcutaneously, cancer cells were found in most organs during the growth of the tumor tissue, but removal of the cancer resulted in a reduction of cancer cells in all tissues except bones where osteoblasts seemed to induce dormancy, possibly through an adhesion kinase (127). The latter study indicates a possible treatment option in patients with bone metastases due to prostate cancer.

**Table 1:** Common features in cancers with late metastases.

Cancer	Cell of origin	Stimulus	Late recurrence	Bone metastases
**NE tumors**	NE cells	Specific hormones	Yes	Yes
**Breast**	?	Estrogen	Yes	Yes
**Prostate**	?	Androgens		Yes
**Kidney**	EPO cell	HIF	Yes	Yes
**Skin**	Melanocyte	UV irradiation	Yes	Yes

NE, neuroendocrine; EPO, erythropoietin; HIF, hypoxia-inducible factor.

Finally, single-cell genetic analysis of cancer cells is gaining popularity and may improve the identification of cells of origin with greater certainty as well as give information for individual treatment. In a lineage analysis in a mouse model, basal epithelial cells were shown to express great plasticity, and both basal and luminal cells were shown to develop into cancer (128).

## 8 Conclusion

To understand carcinogenesis, it is important to identify the cell of origin and its growth regulation, which also makes it possible to tailor treatment. It is obvious that differentiated cells secondary to long-term stimulation may develop into cancers. The role of cancer stem cells in carcinogenesis has often been explained by their ability to stop proliferation but at a later stage are highly proliferating. There is, however, a lack of experimental evidence supporting this idea. Conversely, differentiated cells with low adherence occurring spread among other cell types in the normal situation may not need great changes before being able to metastasize at a stage of minimal alterations of other functions, including cell division. Such metastasized slow-growing cells will naturally need a long time to develop into a macroscopic tumor. No common new mutations between the initial tumor and the late metastasis, in contrast, indicate that the tumors developed separately due to a common carcinogenic cause.

## Author contributions

HW: Visualization, Writing – original draft, Writing – review & editing. GS: Visualization, Writing – original draft, Writing – review & editing.
